# Remarks on Some of the More Prevalent Diseases of Ohio in 1848

**Published:** 1849-06

**Authors:** John Dawson

**Affiliations:** Jamestown, Ohio


### WESTERN JOURNAL

OF

### MEDICINE AND SURGERY.

JUNE, 1849.

ART. I.—*Remarks on some of the more Prevalent Diseases of Ohio in* 1848. By JOHN DAWSON, M.D., of Jamestown, Ohio.

Having a little leisure at present, we embrace it to present a brief account of the diseases with which our region, during the last year, has been visited; and, in doing this, our remarks will be chiefly confined to some of the more prominent of these affections.

During the season, we experienced nothing of an unusual character, as regards the quantity of rain, temperature of the weather, etc. The spring was warm, with perhaps rather more than the usual amount of rain. A pleasant summer followed, the temperature of which was at no time high. The transition from summer to autumn, and from autumn to winter, was gradual, being attended with nothing that seriously interfered in any way with the general health. The crops, so far as wheat, oats, and corn are concerned, were abundant. The same is true of fruit. We here had the largest crop of apples, cherries, plums, &c., that has occurred for years.

With all of these favorable circumstances, it might be supposed that we have been almost, if not entirely, exempt from disease of every kind. But we have not. There are some diseases which we meet with more or less every year; and there are others of which have but an occasional prevalence. Among the former, we find the various forms of disease usually alleged to be of malarious origin, such as bilious remittent fever, intermittent fever, neuralgic pains, &c.

*Bilious Fever.*—This complaint has not prevailed so extensively in this part of Ohio, nor, as far as we have learned, in any other part of the State, as in former years. Comparatively speaking, we treated but few cases during the last season; some of these, indeed the most of them, were mild. We saw, however, a few cases of a very malignant character, so much so, that medicines seemed to possess no control over them.

Our treatment has varied according to the form of the complaint and its complications. Regarding it as owing its origin to the same cause as that producing intermittent fever, only operating in a more concentrated form, we have adopted a corresponding system of medication. The most obvious indication has seemed to require something to neutralize or destroy the virus. After clearing the first passages with cathartics or emeto-cathartics, it has been our practice to trust the cure of uncomplicated cases to quinine. With respect to the periods most proper during the course of this fever, for the introduction of this drug into the system, there has been some variety of opinion. All agree that when a remission can be obtained that is the most proper period. No one, however, should conclude from this that, in those cases presenting a very formidable array of symptoms, and not marked with any very appreciable remission, the drug should be withheld. Under such circumstances we have administered it, and so far are well satisfied that it is better to do so, at these inauspicious times, than to withhold the drug for days, and sometimes weeks, waiting for a remission.

As there is still some diversity of opinion upon the propriety of the quinine plan of treating the disease before us, we will offer a few thoughts in its defence. Without summoning the success of the practice in our own hands, as applicable to all cases that we suppose can be cured by any course of treatment, we believe its propriety may be established from a class of facts touching its identity and mode of origin with intermittent fever. Both diseases prevail in the same localities, and the circumstances, meteorological or otherwise, which favor the prevalence of the one, favor the prevalence also of the other; both are characterized in the onset by the same symptoms; both take life in about the same way. Remittent fever, as is well known, prevails sometimes without any appreciable remission. The same group of symptoms we see, in another case, connected with a decided remission; and what is more common than to see the disease, in cases where there has even been no treatment whatever instituted, terminate in "*chills*," showing clearly that it passes by a series of gradations into intermittent fever. On the contrary, if we are to believe the testimony of physicians, intermittent fever will occasionally become *continued*, characterized by the usual phenomena of continued fever. With, then, as far as anything is known of either of the complaints, an identity of origin and pathology, what is there unreasonable in supposing that they have an identity so far as the effects of remedies are concerned? Further, this matter is sustained by a great number of medical gentlemen residing in highly malarious districts.

*The Biliousness of Remittent Fever.*—This is a circumstance which has formerly entered largely into our views in almost every case of autumnal remittent fever. Whether it is real or only imaginary, is a question of no small importance to the practitioner. Writers, not regarded as being at all obsolete, and, we may also add, some teachers of the present day have, somehow or other, succeeded in making the impression that most, if not all cases of the complaint before us, are connected with pretty prominent derangement of the liver, and hence the currency of the name "*bilious*" fever. That the liver, like other important organs of the economy, undergoes alterations both of function and structure, is a position the integrity of which will never be assailed by ony one possessing but a limited experience. That, however, it is so much involved in evcry case, as to give character to the complaint, is a view which derives its origin and support from a source of kindred character with that of the distinguished Frenchman, who makes gastro-enteritis the prevailing lesion in all cases of remittent fever. Derangement of the liver we regard as a complication; as something that may or may not supervene during the progress of the malady, and not as one of the indispensable elements, which go to make up the series of morbid actions of which the disease is composed. Of itself, derangement of the liver is a disease, and one too of very frequent occurrence in malarious districts. As a consequence, it is frequently associated, not only with remittent fever but also with other diseases peculiar to such localities, masking sometimes the character of the disease with which it is connected in such a manner as to appear to be decidedly the most formidable and important lesion present. Without doubt, it is this circumstance, more than any other, which, in the treatment of autumnal remittents, has given rise to a system of medication the principal indication of which is made to consist in the use of medicines addressed to the liver. By making such an indication the principal one, it is that the error is committed. In our view, instead of administering mercurials for a number of days and nights in succession, in order, as is imagined, to restore the secretions, a better practice consists, after the first passages are cleared efficiently, in the administration of quinine. This course will arrest the fever, after the subsidence of which the secretions of the liver are easily managed, much more so, indeed, than when the fever was in full possession of the system. If we are not mistaken, this plan has several advantages. It saves the patient's time, his money, and the wear and tear of his system by medicine. It may be replied here, that there are some cases so decidedly *bilious* that quinine will have but little, if any, effect upon them until they are pretty well mercurialized. Readily we grant the truth of this. Just such cases we have witnessed. We have also seen other diseases, intermittents for example, so intimately associated with morbid conditions of the liver that policy suggested the propriety of conciliatory measures towards this organ before making an attack elsewhere. By us, however, such cases have been regarded as exceptions to what generally obtains.

Another view of this inquiry, going to favor the position that biliousness is but a complication of our autumnal remittent fever, will be found in the fact, that in many of the very worst cases of the complaint, those usually termed malignant, derangement of the liver is not at all apparent. The disease seems to take life by a series of morbid processes, in which the liver seems but slightly to participate. After the stage of incubation, in such cases, a chill, or perhaps rigors are developed, followed by fever of the synochus grade, nausea, vomiting, diarrhea, intense pain in the head and back; then follows a sinking sensation in the epigastrium; frequent thin watery discharges from the bowels; increased thirst; dryness of the tongue and throat; incoherence, and other evidence of disturbance of the brain; small, frequent pulse; cold clammy condition of the surface; cold extremities. The fatality of such cases has always seemed to us to be due to a very violent impression of the virus upon the stomach and bowels; for, at the bedside, it has seemed impossible to resist the conviction that these organs were the seat from which morbid actions were radiated to other parts of the system. By no means, however, do we wish to be understood that such cases are entirely free from trouble in the liver. Indeed, no malady, with the array of morbid actions to which we have referred, can be supposed to exist for any length of time without interfering more or less with the functions of an organ holding such relations to the system as the liver. What, nevertheless, we design to say is, that lesions of the liver, whether of function or structure, bear no comparison to the alterations going on in other parts of the system.

*In what manner is the liver usually affected in remittent fever?* Is the flow of bile *increased* or *diminished?* Or is the trouble due to *morbid bile?* Diversified are the opinions of physiologists concerning the purposes for which the liver secretes the bile—what functions it performs in the process of digestion—whether it is intended merely for excretion; or, as has recently been affirmed, is returned from the intestinal canal again into the organism—are questions which have not shown themselves amenable to any of the researches of physiologists or chemists however profound. And yet it would seem necessary to have some correct conceptions of these questions, in order to have a starting point, from which to deduce rational conclusions with reference to the pathological conditions of the organ.

Tiedemann and Gmelin regard the function of the liver as being supplementary to that of the lungs—to remove hydro-carbon from the system. They urge that warm-blooded animals have the liver of a size that is inversely proportionate to the size and perfection of the lungs. Thus, animals which live in the open air have large lungs and small livers, while those which live partly in the water, with a very imperfect apparatus for the decarbonization of the blood, have a very large development of the liver. They also suggest that, in pneumonia and phthisis, the secretion of bile is increased. As further going to show that the biliary secretion is in a great measure supplementary to the decarbonizing process of the lungs, we are referred to the physiology of the fœtus, in which the liver is larger, proportionally, than in the adult, and in which the bile is copiously secreted.

To some extent the views of Liebig are analogous to those to which we have adverted. He supposes that those compounds which contain the carbon of the transformed tissues are collected in the gall bladder in the form of a compound of soda, the bile, which is miscible with water in every proportion, and which, passing into the duodenum, mixes with the chyme. The parts of the bile which, during the digestive process, do not lose their solubility, return into the circulation in a state of extreme division. As proof of the capability of the large and small intestines to absorb bile, we are referred to the circumstance that bile, mixed with enemata and injected into the rectum, quickly disappears, and also to the feces of the carnivora, which it is said contain no bile, the whole having been reabsorbed.

While both of these theories regard the liver as an organ the primary and principal use of which is to separate carbon from the blood, the former considers the bile, or the carbon it contains, as an excrement which passes off with the fæces; whereas, the latter supposes it is principally returned from the alimentary canal into the circulation. How now are we to reconcile, by either of these theories, the manner in which disease is produced by an *increased* or *diminished* flow of bile? If, as one of them presumes, the separation of the bile from the blood is a process perfectly excrementitious in its character, there is reason to suppose that any agent which interferes with this process gives rise to disease in the same way that the obstruction of any other emunctory would do. The elements of which the bile is composed would be retained in the circulation, acting, of course, as an irritant, and thereby producing alterations on the internal coats of the arteries, or perhaps fever. But, if children can live four months in the enjoyment of apparent health, without having any apparatus by which this alleged irritant can be separated from the blood, as Blundell found to be the case with two children, such ways of accounting for the origin of diseases may have as much ingenuity about them as truth. The other theory, which teaches that the bile is not merely an excrement, but is returned into the circulating mass from the intestines, it seems to us, is more plausible, though it is not without its objections.

Speaking on a kindred subject, Dr. Jas. Johnson refers to what he calls a cutaneo-hepatic sympathy—a sympathy between the liver and skin—which, in warm climates, gives to fevers, as well as other diseases of a bilious char acter, in consequence of there being an increased flow of bile. Other writers, whether they avow the connection between the liver and skin or not, as suggested by Johnson, nevertheless agree with him in the fact, that it is an excess of the biliary secretion which usually complicates the diseases of summer and warm climates. According to this view, mercurial purges owe their efficacy, in remittent fever, to the property they possess of either restraining the action of the liver, that has already been goaded to excess, or simply to their purgative property in removing effete bile from the alimentary canal. If to the former, where the propriety of the course so usually practised of administering them in other diseases of alleged torpidity of the liver? if to the latter, in what respect are they better than other efficient purgatives?

Functional or structural disease of the liver may, of course, give rise to a depraved character of the secretion. With reference to this matter, there has been, so far as we are acquainted, no observations made on the bile of persons dying from remittent fever. The bile in some other complaints has not been overlooked. Forget states that in persons suffering from dropsy it is *thinner*, while in those laboring under a diseased liver it is thicker than in the normal state. In persons that died of cholera, Phœbus found the gall bladder unusually full, sometimes to excess, and the bile rather dark colored; and Heraman found that in the same disease it contained an excess of resin. In his researches on the bile of a dropsical boy, Lehmann found a large quantity of hydrosulphate of ammonia, a circumstance which, in other cases, did not occur when the bile had been kept for some days. Simon and Bizo examined the bile of cases which had been affected with icterus; the former obtained a very small quantity of viscid, dirty, yellow fluid, from which alcohol precipitated mucus and albumen; the latter found the bile to be of a dark red color, thick, of a nauseous, but not bitter taste, with an odor resembling that of putrid fish, and holding in suspension red and black particles. Chevalier states that the bile in the case of a man who had died of scirrhous pancreas, and who was jaundiced, was of a pale green yellow color, had a putrid odor, an alkaline reaction, and a faint slightly saline taste. The last named author, as well as Chevereul, found great departures of the bile from the healthy standard in phthisis and syphilis; and Thenard, in cases of fatty degeneration of the liver, noticed a diminution of biliary resin, the bile appearing as a mere albuminous fluid.

Such facts as the above we think are sufficient to excite some inquiry with reference to *morbid bile*, as one of the circumstances which may be connected with the biliary derangements incident to our autumnal fevers. If the bile is not an excrement, as has formerly been supposed, but a recrementitious substance, the principal elements of which are designed to be retained in the system as inservient to the process of respiration, how easy is it for the mind to recognize the want of balance in the elements of which the fluid is physiologically composed, or the introduction of new elements into its composition, as agents that may exercise some influence in giving rise to that group of symptoms by which we are enabled to recognize biliousness?

To the phenomena generally regarded as being evidence of biliary derangement, it might not be improper here to advert. No one perhaps attempts to make out a biliary complication without referring to the appearances of the tongue, skin, conjunctiva, and character of the stools.

The tongue may or may not be of service in the diagnosis, owing to the fact, that in different individuals in a state of health, it varies as it regards its form, its color, and the coats with which it is liable to be affected. On the contrary, we frequently see the tongue remaining in a physiological condition, while some serious disease is present in the system. Usually it is supposed that a yellowish or brownish appearance of the coat indicates biliary derangement. This may or it may not be true. In recent cases, the appearance may be owing to the presence of something in the food, or something in the medicines administered. In cases of longer standing, it may be owing to the foul, dirty secretions with which the mouth may happen to be affected. What more common than to see a brownish dirty tongue in typhus, which, perhaps, owes its existence to the same cause which affects the teeth with sordes. Hence it is only by taking the appearances of the tongue in connection with other evidences of hepatic lesion, that we can derive from them any consequence in the diagnosis.

On the color of the skin and conjunctiva very much reliance has been placed. But of the manner in which this color is imparted to the skin, and of its true import, we know but little more than what is sufficient to excite inquiry. Generally it has been supposed to be due to the presence of bile in the blood. According to the experiments of Orfila, Collard de Mortigny, and Clarian, on the blood of persons who were suffering from icterus, it was found to contain no other constituent of the bile than its pigment or coloring matter, lately named biliphein. Lessaigne and Thenard declare that they have been unable to detect in the blood of such persons any trace whatever of the bile. The most general opinion, however, is that the yellowish color of the skin is due to the presence of the pigment in the blood, and some think to this alone. Taking this for granted, another question arises as to its import. Is it connected with an increased or diminished flow of the bile? or with a morbid state of this fluid? Becquerel and Rodier both observe that in icterus there may be a continued secretion and flow of bile, or there may be perfect retention arising from biliary calculi, &c. No particular modification was observed by them in the blood in the first case, while in the second there was an accumulation of cholesterin and the other fatty matters. When the flow of bile is copious, much more being secreted than in a healthy state, of course it may contain more than a corresponding proportion of the pigment, which may give color to the skin by its abundance in the system. When, on the contrary, the ductus communis choledochus, from any cause, becomes obstructed, even should there be a deficient amount of bile secreted, it being unable to pass off by the natural channel, must, as a consequence, remain in the blood to color the skin. We may suppose, therefore, that the icteric appearance denotes trouble of some kind or other; but of the character of this and its gravity in a given form we know almost nothing. How frequently do we see patients affected with yellowness of the skin, which makes its appearance during convalescence from some complaint not apparently interfering with the functions of the liver, and after continuing awhile subsides without drugs of any kind being used? The icterus connected with disturbances of the digestive process, and that frequently noticed in very young children are also of difficult comprehension, and induce us to believe that those who have investigated the matter most will be the most careful about giving any very decided opinion.

The appearances of the stools are usually regarded as evidence of a very important character in making out biliary derangements, whether existing as a primary disease or as a mere complication of some other. The brown or yellowish cast of the feces of a healthy adult is usually attributed to the presence of bile. Berzelius, who is the highest authority on this subject, found in one thousand parts of fresh human feces only nine parts of a substance similar to bile. Now, as Liebig suggests, five and a half ounces of feces, the usual weight daily discharged, would contain only 21 grains of dried bile, equivalent to 219 grains of fresh bile. A man, however, secretes daily from 9.640 to 11.520 grains of fluid bile; that is from 45 to 56 times as much as can be detected in the matters discharged by the intestinal canal. From this it appears that the most of the bile is absorbed, leaving, in fact, but nine out of one thousand parts to impart the characteristic appearance. Whether now the appearance is due to this small quantity, and to this alone, future research can only determine. In a state of health, the color and consistence of the stools vary according to the kind of food used. A person living to a considerable extent on rice will, as we recently witnessed, have stools that are much fairer than one who uses a good deal of bran bread, or some other article of a dark color. And during the summer and autumn, when fruits enter largely into the food, the evacuations are not only thinner, but present a greater diversity of hue than under other circumstances.

Various are the opinions with reference to the cause of the green stools, called, by Kraus, "*calomel stools*," which sometimes follow the use of this preparation in the disease of children. Some suppose that they arise from the action of the medicine on the liver, though Kraus himself thinks that they are owing to the action of calomel on the milk contained in the alimentary canal. Zeller contends that the color of the stools is caused by alterations produced in the condition of the blood. Bird published an analysis which he made of one of these green evacuations. The specimen examined was passed by a hydrocephalic infant while under the influence of mercury, and the composition of the fluid part of the green evacuation is thus expressed: Water 900.00; biliverdin, alcohol extract, fat cholesterine, with traces of bile 24.50; ptyalin, aqueous extract colored by biliverdin 11.25; mucus, coagulated albumen, and hematin 56.00; chloride of sodiam with traces of tribasic phosphate of soda 5.50; tribasic phosphate of soda 1.75; peroxyde of iron 1.00. Kersten denies that the greenish tint is dependent on the presence of bile, but ascribes it to the green sulphuret of iron. Berzelius says he never suspected that it arose from sulphuret of iron, but regards it as being due to the black oxyde of iron. Bley supposes that it owes its existence to the sulphuret of mercury. Our own observations lead us to the conclusion that the greenish stools of children are sometimes the result of processes which go on in the alimentary canal independently of the agency of any foreign substance whatever; for we think we have frequently recognized them in cases in which no drug of any kind had been administered.

Simon endeavored to ascertain whether the very dark green bilious stools of adults, which, in certain morbid conditions of the system, follow the administration of calomel, were due to the bile and its pigment. The fifth stool that was passed, after the administration of a large dose of calomel, was made the subject of the analysis. Bilin with bilifellinic acid and biliverdin were found in large quantity, and by digestion with sulphuric acid the bilin became converted into biliary resin. He adds, after various attempts that he made (by Smithson's method) to detect mercury in calomel stools, the result proved unsuccessful. That calomel, however, does produce alterations in the appearance of the discharges from the bowels, has been rendered certain by the experiments of Annesley, who found that calomel administered to dogs gives the discharges a dark color.

As a general rule, clay-colored feces are regarded as evidence of a diminished flow of bile, or perhaps a want of balance in the constituents of which the bile in a normal state is composed. Usually, but not always, persons affected with jaundice have clay-colored stools. Piercy analyzed the feces of a young lady affected with jaundice, which, instead of being clay-colored, were brown. Indeed, it is not uncommon to find bile in the feces while the skin and eyes are yellow. This is thought to depend upon the circumstance, that some branches of the hepatic ducts are obstructed while the others are free; and thus the bile that is secreted is in part conveyed into the intestines, and in part reabsorbed. While, therefore, white or clay-colored stools may safely be regarded as depending on the absence of bile, biliary obstructions, as we have just seen, may exist without imparting any such character to the feces.

We have now reviewed the more prominent phenomena usually depended upon for the purpose of diagnosticating a case of biliary derangement, such as we are likely to meet with in the treatment of autumnal fevers; and we believe that no one, who passes over the subject carefully, can come to any other conclusion than that we are yet merely upon the threshold of knowledge with reference to the whole matter.

Before dismissing remittent fever, we have a word or two to say with reference to its tendency to terminate in "*the chills*." Formerly this was regarded as being a salutary result, and as a consequence measures were sometimes put in requisition to bring it about. That it should still be so considered, in many cases of the disease, is a position from the correctness of which we by no means dissent; for the practice of converting a severe disease into one of less dangerous character, is perhaps as reasonable as any thing connected with the allopathic mode of practice. By no means, however, does it follow that such is a correct mode of procedure in all cases. When once we are satisfied that the exchange of one form of disease for another would probably increase the aggregate amount of suffering that the patient would have to endure, propriety would suggest that the exchange should not be made. For ourselves, we believe that, so far as converting an ordinary case of remittent fever into the chills is concerned, this is the case. As a general rule, remittents are of short duration and easily managed. Can this be said of the chills by which they are so frequently succeeded? After many cases of fever are cured, during the summer and fall, the chills set in, and in spite, very frequently, of remedies, keep the patient sick during the winter and part of the spring. To such an extent have we witnessed this for some years back, that instead, in the majority of cases, of adopting any measures to convert the one form of disease into the other, we usually try to avoid such a result. With reference to the best means of doing this our experience is not at all extensive. We are aware, however, of one thing, and that is, that it, in very many cases, is difficult. The very means we often institute for the cure of a patient affected with a bad form of fever, such, for example, as bleeding, depleting, purges, &c., prepare the system for the advent of the chills, hence it is that such cases, more frequently than any other, terminate in the chills. Although, in these cases, we are unable to avoid the result, the great majority require no such treatment. They can be cured without blood-letting or any purgation, more than a clearing once or twice of the bowels, as we have shown in a previous part of this paper. In addition to the avoidance, in all ordinary cases, of a decidedly atonic course of treatment, something may also be done by guarding the period of convalescence with the administration of quinine at stated intervals. It is thought, by those having an extensive acquaintance with the habits and habitudes of the disease, that it shows a tendency, after once being cured, to return on or about every seventh day, and it is during these hebdomadal paroxysms that the form of the complaint is most likely changed into that of the chills; and hence the propriety of anticipating these periods with the administration of quinine.

*Intermittent Fever.*—The prevalence of this disease has been much greater in former years than during the past; and what cases did occur had nothing about them of much interest, except that, in a few of them, the paroxysm seemed to be incomplete. We witnessed several in which the chills made their appearance at pretty well defined periods, being followed with a high fever, but *no sweating*

*stage*. Instead of the sweating stage, there were violent neuralgic pains. Sometimes they were seated in the head, and gave rise to the most intense suffering that we ever noticed in cases of fever. They were the most protracted of any that we treated during the season. Quinine, in the doses that cured other cases of the complaint, made but little or no impression. In one case, which was the worst that came under our care, we persisted in the use of quinine for about three weeks before the disease was arrested. We commenced with the ordinary doses, but ended with larger ones; and, although the patient recovered, we were not able to attribute it with any great confidence to the quinine. No prominent derangement of any of the secretory organs was observable, except the skin, which remained rather harsh during the most of the course.

With respect to the treatment of intermittents no one feels much interest, owing to the fact that we are about as near perfection as can ever be expected. But *the best mode of keeping the complaint cured* is now decidedly the most important matter that should engage the attention of the practitioner. Quinine will cure the disease, but what is the best calculated to keep it cured? We have experimented with a number of the drugs most likely to accomplish this object; but as yet we are not able to give a very decided opinion upon their relative merits. The administration of quinine and carbonate of iron, just before the periods of the expected recurrence, and persisted in for some time, will do as much in keeping the disease from returning as any thing we have used. Some practitioners, believing that the malady, after being completely cured, has a greater tendency to relapse on or about every seventh day, than at any other times, address their prophylactic measures to these periods, and they think with decidedly more success than in attempting to forestall or anticipate the paroxysms by administering medicine every day or every other day. As we design preparing a paper especially on intermittent fever before long, we will waive any further notice of the subject at present, and make a few passing remarks upon some other diseases with which we have been visited. One of these is—

*Scarlet Fever.*—In the latter part of fall, this disease made its appearance in our region, and is yet prevailing. As far as it has come under our observation, it has in the general been mild. This, however, as report says, is not true of some regions not far distant. There, it is said to be prevailing with considerable mortality. As far as we have noticed the eruption, it has usually been complete. Nor have the fauces, as a general rule, been severely affected. That variety called *scarlatina maligna*, and which is alleged to be identical with *putrid sore throat*, it is worthy of remark, has not been observed since the epidemic made its appearance. Nor have we ever, so far as we now recollect, known it to prevail with the other varieties. So far the fever connected with the present epidemic shows rather a tonic condition of the general system, the very reverse of what obtains in cynanche maligna. Indeed, the more we see of the two diseases the more we are convinced that, instead of being identical, as is alleged to be the case by some modern writers, they are separated from each other by as many peculiarities touching their origin, pathology, and treatment, as are usually found to exist between different diseases.

With respect to the best mode of treating scarlatina there is still some diversity of opinion. Believing that the complaint has a certain series of changes through which it will pass, we have regarded all attempts to stop it, or even abridge it, as being productive of no benefit whatever, but, on the contrary, as calculated to convert mild cases into such as it will sometimes be found difficult to cure. During the present epidemic we have not used the lancet in any case, notwithstanding we have witnessed an array of symptoms which seemed to suggest to us in very strong terms the propriety of a loss of blood, and which, if they had been connected with almost any other disease, would have had the efficaciousness of that measure tested; yet our views of the pathology of the complaint induced us to withhold a procedure, the principal effect of which we believed would be to embarrass the efforts of the system in the elimination of the virus. Cautious also has been our use of purgatives. Those of a character decidedly mild, such as hydrargyrum cum creta, rhubarb, or an infusion of senna, has always seemed to us to be more in harmony with the irritable condition of the mucus membrane of the first passages, than others, the impression of which is attended with some considerable degree of severity. Indeed, during the use of purgatives of any kind the state of the bowels should be closely observed, in order to guard against the production of diarrhea, a circumstance usually of very serious import, and so often connected with fatal cases. Why is it that so many cases of this disease recover when treated with domestic remedies? In answer to this question it may be submitted—1, that mild cases require little or no interference of any kind; and will recover, unless they are embarrassed by too much officiousness—2, that purgative drugs are in less favor with the community as a remedy than tisans, mucilaginous drinks, and, as a consequence, when they do attempt medication of any kind, it is with a class of articles that the closely observing physician would, perhaps, rely upon in similar cases. Local medicines in point of importance hold a high place in the treatment of almost every case. In cases where the surface is hot and dry, the application of water, or water and vinegar, of a temperature below that of the skin, will exercise not only a very salutary influence over the feelings of the patient, but will do much towards allaying arterial excitement and bringing the functions of the skin into a healthy state. Equally valuable is warm water when the fever is connected with an atonic condition of the system, attended with an imperfect eruption. About the throat and nasal fossæ the defluxions are sometimes very urgent, and in such conditions the application of emollient cataplasms will scarcely ever fail to soothe the sufferings of the patient. A case occurred a few weeks since in a boy of some fifteen years of age, attended in the onset with high fever, urgent thirst, violent pain in the head and back, delirium at times, and occasional diar rhea, and a condition of the throat and nasal fossæ very much swollen. We strongly doubted the recovery of this case. The array of symptoms was such as inclined us to the opinion that it was one of those cases that generally go to a fatal issue under any or all plans of treatment. During our absence on one occasion, and while the patient was in the worst stage of the complaint, a *tar plaster*, made of very thin tar, was applied so as to encircle the entire neck. On our return we found the patient better—left the tar plaster in its place, and, to our sur prise, he recovered. Was this a coincidence, or should the salutary change be ascribed to the agency of the tar? Affected in a similar manner and with just such a group of symptoms, patients, according to our observation, are apt to die; and nothing could be discovered in the case before us, previously to the application of the tar, that would justify the anticipation of any other result. We therefore concluded to give the measure the benefit of further trial; and although none of the cases in which it was afterwards used were so severe, its influence seemed sufficiently positive to create a favorable impression of its efficacy.

There is something in scarlatina, like other diseases of the same class, which teaches the practitioner some severe lessons of mortification. While he finds many cases do better without than with drugs, there are others that seem to bid defiance to all the resources with which he is acquainted, and go to a fatal issue without apparently having their symptoms in the least manner mitigated. It is true, nevertheless, that between these mild and malignant cases there is a class in which well directed efforts may turn the scale in the patient's favor. But even here the utmost skill is often requisite to prevent remedies from *coinciding* with the disease.

*Pneumonia.*—Into any thing like a minute account of the disease which stands at the head of this paragraph, we do not expect at present to enter. But, as there is more or less of it witnessed every year, we think it not out of place, in a communication like this, to give it a passing notice. During the months of winter and spring, more than in any other parts of the year, does the disease prevail in our region. It is more common some years than it is in others; and it was the opinion of a very close observe in Ohio that its prevalence had a very intimate relation to the temperature of the winter months. When this is high, but little, comparatively, of the complaint makes its appearance; when low, it makes up no small proportion of the sickness and mortality incident to the season. It is not, according to our observation, of the most frequent occurrence during the presence of the coldest weather; but while the transition from winter to spring is going on the greatest number of cases are developed. While all classes are perhaps equally affected, the old are certainly the most liable to suffer. No one complaint, we are satisfied, carries off so many aged persons in this region as the disease before us. Two causes, probably contribute to this result—the inability of old persons to bear a low temperature, and the greater tendency there is in the inflammatory action to diffuse itself through every part of the lungs. Among children our opinion is that it is less fatal than with any other class. Nor is it with children so frequently idiopathic, being wholly engrafted on some other disease, as catarrh, influenza, measles, etc.

With respect to our method of treating pneumonia, it has been governed not by the numerical statistics, but by attention to the age of the patient, the constitution, the cause of the disease, its character, &c. Aged persons, as far as we have known it tried, bear the loss of blood badly. A few years since, it became proverbial among the people that all the old persons who were bled died. Although this was not true, we nevertheless believe that mischief is usually the result of bleeding this class of persons, unless great discrimination is observed. Stimulants and diaphoretics, local bleeding and blistering, are measures better adapted to a large proportion of these cases. Connected with the constitution one of two condi tions of the system always obtains, the tonic or the atonic. In young persons, with good habit, and a decidedly firm state of the organism, general bleeding will accomplish every thing alleged concerning it by its warmest friends. But even here we have always inclined to the views of Gregory, that early bleeding, and that carried to the point of syncope, will exercise a more salutary influence over the morbid action than a very frequent repetition of the measure, and the abstraction at each time of smaller quantities. In atonic states of the system the value of the measure is much less evident. One bleeding may frequently be tolerated, but a repetition of the measure has, according to our observations, scarcely ever failed to lessen the patient's chance of recovery. To cupping, blistering, tartar emetic, lobelia inflata, and diaphoretics, under such circumstances, we should rather trust. Malarious influences sometimes modify the powers of the constitution in such a way as makes it necessary to make essential departures from the course usually found successful. Dr. J. Johnson, of Laramie, Ia., since we commenced this communication, informed us that, in his region, it was found impossible to treat pneumonia, in many instances, successfully without quinine. After the usual remedies, patients would linger until this drug was used. In children, the disease is frequently engrafted on some other complaint, as, for example, pertussis or the measles, or perhaps is a sequel of catarrh. Cases of this character neither call for nor can they be successfully treated by the usual routine practice. Besides the general remedies which each individual case seems to require, it has been our practice in many cases to use, as a local remedy, cataplasms sufficiently large to envelop the chest and part of the abdomen. Children, particularly young children, bear blistering and cupping, as all know, with a great deal of inconvenience; and the good which they are frequently supposed to do is in a great measure counterbalanced by the irritation which they create. In some instances where the whole system has seemed to be in a violent state of irritation, we have added anodyne substances, such as hops, and, in the absence of this article, boiled onions, to the poultices, and it has generally seemed to us with benefit.

With a brief sketch of a certain disease not at all connected with any of those previously noticed, but one that is occasionally met with in going the rounds of treating disease, and one, too, which will give the practitioner about as much trouble as any that we have noticed, we will conclude this communication, so far as a notice of disease is concerned. We allude to

*Colica Menstrualis.*—From a case of this troublesome complaint we have just returned; and, owing to some peculiarities connected with its progress and treatment, we have thought it not out of place to give it a passing notice.

The lady to whom it occurred is about thirty years of age, is the mother of some four or five children, and has the appearance of enjoying good health. For the last seven or eight years she has been somewhat irregular in the menstrual function, and at such times she has been liable to attacks of colic, the pains of which have always seemed to be seated in the stomach, radiating from this point towards the chest or lower bowels. The last attack came on her during the presence of the menstrual secretion, and a short time after she had been eating an apple. Several things were administered before we saw her, the effects of which produced copious emesis. We commenced the treatment with opiates in large doses, and continued them until we had administered about two drachms of laudanum and six or eight grains of opium. The vomiting and pain in the stomach persisted for some length of time, but by using fomentations to the epigastrium in conjunction with the opiates, a temporary respite from the most urgent suffering was obtained. This, however, lasted but a few hours, when pains as violent as ever returned. Foiled in our efforts to destroy the pain with the preparations of opium, purgatives were administered, and with the first evacuations the symptoms were strikingly mitigated, and by maintaining a lax state of the bowels for a couple of days the patient was cured.

This, with other cases of the same character which we have noticed within the last ten years, has interested us not merely because they have been characterized by an uncommon amount of suffering, but on account of the treatment which proves most successful. Opium and its preparations, which are so frequently and so successfully invoked in almost every variety of cramp and spasm, seem to have no value whatever. In every case we have treated, they have been first employed, and that, too, in connection with fomentations, rubefacients, sinapisms, and other auxiliaries; and so far we have been disappointed. Brisk purgatives, however, have never disappointed us. In every instance in which they have been used, an amendment has been perceptible as soon as they took effect on the bowels. Their efficacy does not appear to be due to the evacuation of gaseous or irritating matter from the bowels, but to the establishment of the natural in the place of the spasmodic action. For several days after the violent symptoms have subsided, it will be necessary to continue their use. As, however, the mucous coat of the stomach is apt to get very much irritated during the course of the malady, some of the mild articles, as hyd. c. creta, will have the most salutary effect in maintaining a laxative state of the bowels,

Before closing, we will say something with reference to the progress, in our State, of

*Quackery.*—On all sides, the profession in Ohio, at the present time, is beset with almost every form and variety of quackery. Coming from such a diversity of sources, and keeping pace with the lights of science, yea, even outstripping almost every thing useful connected with the improvements of the age, there is good reason for the opinion that we are not yet in the worst stage of the trouble.

In nearly every town and village the stores contain a supply of patent medicines, such as purgative pills, cough drops, ague medicines, liniments, ointments, plasters, specifics for the cure of consumption, for the diseases of the liver, &c. Described in very extravagant and false terms, in large ornamented handbills stuck up in some prominent place in the store-house, and many of them also in the newspapers, the result is, that these drugs are bought and consumed in great quantities, and when the physician is called upon to visit the sick, he not unfrequently finds that his patient has been for days preceding swallowing nostrums that were either inert or coinciding with the malady in their effects upon the system.

The *steam* and *pepper* fever, so far as we are acquainted in the State, has about come to a *crisis*. In the distance, however, new affections, or rather complications of the old, are beginning to show themselves under the various names of "Reformed Botanical Medicine," "Eclectic Medicine," "Hydropathy," "Homœopathy," &c.; and, from present symptoms, the most of those formerly affected with the steam and pepper disturbance are about to take these new troubles in something like the natural way. To drop the figure, the transition from one humbug to another seems much easier than a return to the solid substantial principles of science.

There is another matter which it may not be out of place to notice. We allude to the standing of physicians at present in the estimation of the people. The time has been, it is said, when there was something dignified and venerable associated in the mind with a mere announcement of the name of a physician. Is this the case generally at the present time? May be it is. If so, we should like to see some one well skilled in diagnosis try his hand in making out what state of mind it is which has given rise to the almost universal custom of the people, when they address a physician, of calling him "*Doc.*" Again, in speaking of the attendance of a medical man on his patients, the common phrase has come to be "*waiting on*" the patient. Thus, a very common form of expression, when any one wishes to inquire about the sick is, "*Doc*, how is that patient you are *waiting on?*"

March 12, 1849.

